# Fully inkjet-printed microwave passive electronics

**DOI:** 10.1038/micronano.2016.75

**Published:** 2017-01-30

**Authors:** Garret McKerricher, Mohammad Vaseem, Atif Shamim

**Affiliations:** 1 King Abdullah University of Science and Technology (KAUST), IMPACT Lab, Computer, Electrical and Mathematical Sciences and Engineering (CEMSE) Division, Thuwal 23955-6900, Saudi Arabia

**Keywords:** capacitor, filter, inductor, inkjet printing, multi-jet, radio frequency, silver

## Abstract

Fully inkjet-printed three-dimensional (3D) objects with integrated metal provide exciting possibilities for on-demand fabrication of radio frequency electronics such as inductors, capacitors, and filters. To date, there have been several reports of printed radio frequency components metallized via the use of plating solutions, sputtering, and low-conductivity pastes. These metallization techniques require rather complex fabrication, and do not provide an easily integrated or versatile process. This work utilizes a novel silver ink cured with a low-cost infrared lamp at only 80 °C, and achieves a high conductivity of 1×10^7^ S m^−1^. By inkjet printing the infrared-cured silver together with a commercial 3D inkjet ultraviolet-cured acrylic dielectric, a multilayer process is demonstrated. By using a smoothing technique, both the conductive ink and dielectric provide surface roughness values of <500 nm. A radio frequency inductor and capacitor exhibit state-of-the-art quality factors of 8 and 20, respectively, and match well with electromagnetic simulations. These components are implemented in a lumped element radio frequency filter with an impressive insertion loss of 0.8 dB at 1 GHz, proving the utility of the process for sensitive radio frequency applications.

## Introduction

One of the major advancements in inkjet printing has been the use of ultraviolet (UV)-cured acrylic materials. These liquid inks immediately solidify on exposure to a low-cost UV lamp, and, like acrylic paints, they can be made with vibrant colors. This concept has been extended beyond graphic arts by using hundreds of inkjet nozzles to form fully printed 3D acrylic parts in minutes. Although multi-jet technology is still in its infancy, commercial multi-jet printers are now available^1^. A major advantage of the 3D multi-jet process over other 3D printing methods, such as stereolithography, selective laser sintering, or fused deposition modeling, is that multiple materials can be easily deposited together, just as an inkjet head routinely prints with cyan, magenta, yellow and black. Thus far, multi-jet printing has been restricted to the colorful UV-cured acrylics and wax/gel support materials. Fully printing objects with an integrated high-conductivity metal provides exciting possibilities for additive and on-demand fabrication of radio frequency electronics such as inductors, capacitors, and filters.

To date, there have been several reports of printed objects incorporating metal, focused on RF applications, by using plating solutions, aerosol, sputtering, and low-conductivity pastes^2–4^. These metallization techniques require rather complex fabrication, and do not provide an easily integrated or versatile process. Previous reports have also put the spotlight on other issues with 3D printing techniques for electronics fabrication, such as micrometer surface roughness and low conductivity of the metal^5^. A recent report by Wu *et al.* shows a novel concept of 3D printing, together with syringe injection of a silver paste at 70 °C, to create 3D inductors and capacitors; however, the conductive paste achieves a low conductivity of 2.8×10^5^ S m^−1^, ~200 times less than that of bulk silver^2^. In our previous work, silver nanoparticles were utilized to metallize a 3D printed antenna, but the particles required selective laser sintering to avoid damaging the acrylic material and achieved a conductivity of only 1×10^6^ S m^−1^ (Ref. 6). A major challenge of fully printing electronics is that the high temperature required for the metal is incompatible with the printed dielectric.

Most of the previous work on inkjet-printed Radio Frequency (RF) passives is focused on two-dimensional (2D) inkjet printing of metal on a standard substrate. Redinger *et al.* reported some of the first work on 2D printed capacitors and inductors in 2004, utilizing nanoparticles but achieving rather low quality factors (Q’s) of ~0.5 (Ref. 7). Since then, there have been material and printing advancements enabling 2D printed inductors and capacitors with high-frequency Q’s below 10 (Refs. 8–10). To the authors best knowledge, the combination of these fully printed components to design a GHz lumped element filter has never been demonstrated. Distributed 2D RF filters are generally simpler to print than lumped element filters. Previous distributed filter results have shown inadequate performance, with the insertion loss ranging from 3.6 to 10 dB (Refs. 11–15). One of the best reports has shown an insertion loss of ~0.5 dB at its center frequency (2 GHz), but it utilized inkjet printing along with electroless plating to increase the conductivity and thickness of the metal^16^.

This work provides a process beyond 2D inkjet printing of the conductor on a standard support substrate; the metal is truly integrated into the printed dielectric to build quality multilayer RF capacitors and inductors with crossover interconnects. A major advancement is the low processing temperature (80 °C) of the novel Silver-Organo-Complex (SOC) ink, which overcomes the major challenge of temperature processing compatibility between the printed conductor and dielectric. The SOC ink is cured with a low-cost IR lamp in only 5 min, while providing state-of-the-art conductivity of 1×10^7^ S m^−1^, which is necessary for conductivity-sensitive RF filter applications.

## Materials and methods

### Ultraviolet-cured dielectric ink

**Figure 1:**
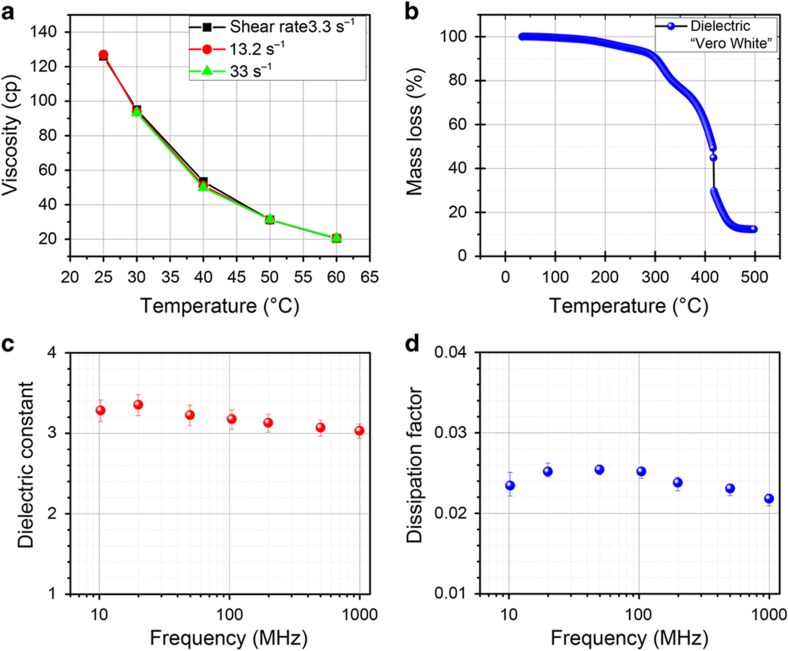
(**a**) Viscosity of VeroWhite dielectric ink. (**b**) TGA of VeroWhite material after UV curing. (**c**) Dielectric constant properties of VeroWhite material after UV curing. (**d**) Dissipation factor. Bars represent the maximum and minimum measurement values of five test samples with the parallel plate method Agilent E4991 and dielectric test fixture 16453A.

Stratasys UV-cured ink is sold under the name Vero and comes in different colors and hardnesses. From the material safety data sheet of VeroWhite, it is composed mostly of acrylic monomers (<30%), isobornyl acrylate (<25%), and various other components, including acrylate oligomers and urethane acrylates^17,18^. Less than 1% titanium dioxide is used for the white color. The photoinitiator is diphenyl-2,4,6 trimethylbenzoyl oxide (<2%), which produces a free radical on UV exposure, initiating the polymer reaction of the acrylic monomers and oligomers to form a hardened acrylic part. The acrylic monomers are liquid at room temperature but too viscous for jetting at 125 cp; see Figure 1a.

Although the UV dielectric ink is tailored for the Stratasys Objet product line of 3D inkjet printers, it was more convenient to print with a Dimatix 2831 printer. The Dimatix printer allows control over nearly all print settings. By using a jetting temperature of 60 °C, the viscosity of the dielectric ink drops to 20 cp, as shown in Figure 1a, allowing for excellent jetting from the Dimatix 10-pL print head. The ink does not show any change in viscosity over the three different shear rate measurements shown in Figure 1a. The average mass of a single drop of the UV-cured ink jetted at 60 °C and a 9-m s^−1^ velocity is 9.6 ng, measured by ejecting five million drops and weighing the total. Throughout this work, the UV-cured ink is printed with two layers at 30 μm, with drop spacing forming 11-μm-thick layers. For curing, it is exposed to 7500 mJ cm^−2^ of 365-nm-wavelength light after printing. After UV curing, thermogravimetric analysis was completed to understand the thermal limits of the material; see Figure 1b. The TGA shows that there is negligible mass loss up to 150 °C; however, the material specification sheet reports the glass transition for the material ~47–53 °C, and it was found experimentally that there is extreme warping of the material over 80 °C (Ref. 19). Therefore, it is necessary to have a conductor that can be processed at temperatures under 80 °C.

To design radio frequency components, it is important to know the dielectric properties. From the parallel plate measurements (Figures 1c and d), the VeroWhite material has a dielectric constant of ~3.0 and a dissipation factor of ~0.02 up to 1 GHz. The dissipation factor of the material is rather high and can cause attenuation of the RF signal; however, FR-4 also has a dissipation factor of 0.02 and is the go-to material for low-cost RF applications^20^. Overall, this material is adequate for many RF designs but does have appreciable dielectric loss, and it has limitations in terms of temperature range (<45 °C) that should be respected.

### Infrared-cured silver-organo-complex ink

The SOC ink utilized in this work has been developed in-house to overcome the issues with conventional metal nanoparticle ink. While silver nanoparticle ink has been widely investigated and is available commercially, it has a complex synthesis protocol, high cost, and high sintering temperature (>150 °C), and exhibits particle aggregation, nozzle clogging, a poor shelf life, and jetting instability. Through the use of smaller nanoparticles (~10 nm) and a more robust ink formulation, commercial silver ink performance has improved considerably in recent years. However, in the long term, it is difficult to avoid particle aggregation and precipitation in a nanoparticle system. Organometallic inks are another approach to printing conductive patterns. They contain dissolved precursors of metallic elements bonded with organics (that is, silver acetate, silver oxalate, and copper hexanoate)^21–23^. In general, the organometallic bond is broken, and the organic molecule evaporates away, leaving a metal film behind. In the past, organometallics have been less successful than their nanoparticle counterparts. One issue has been bubble formation, resulting in rough porous thin films as noted by Walker *et al.*^24^

An in-house SOC ink is utilized in this work, and it is capable of producing smooth and dense films; it is stable and transparent, with full details covered in the reference^25^. The SOC ink produces films with an impressive 1×10^7^ S m^−1^, 20% of the bulk conductivity at only 80 °C. Along with the high conductivity at low temperatures, the ink exhibits strong adhesion, long-term stability (>5 months in a print head), and robust jetting performance. Briefly, the ink is based on a silver acetate complex with ethylamine, ethanolamine, water, methanol, and 2-hydroxyethyl cellulose (HEC) (Mw ~90 000), where the HEC acts as both viscosity modifier and adhesion promoter. The fluid parameters and operating points of both the SOC ink and the VeroWhite dielectric ink are given in Table 1. The Reynolds, Weber, Ohnesorge, and capillary number are provided since inks are often mapped by these parameters to fit in a specific jetting space. Although the SOC ink has a lower viscosity than the dielectric ink at 5.9 compared with 20 cp, both inks have good jetting stability and fit within the jetting space outlined by Derby *et al.*^26^

**Figure 2:**
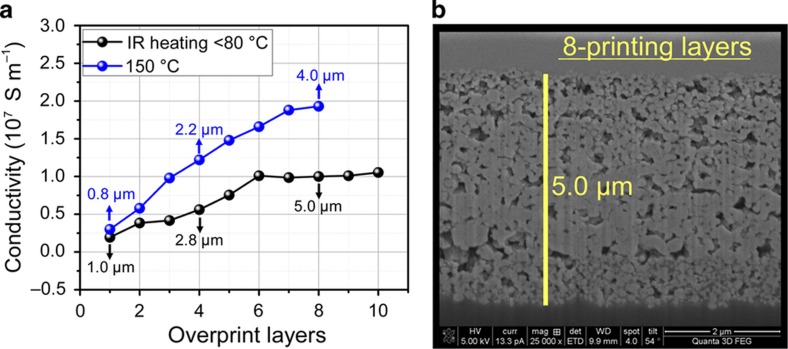
(**a**) Conductivity as a function of overprinting layers at different sintering conditions, that is, thermal heating at 150 °C/30 min and IR heating at <80 °C/5 min. (**b**) SEM-focused ion beam cross-section of 8-layers of SOC ink with 5 min of IR <80 °C treatment after each overprint.

The conductivity of the SOC ink has been tested as a function of layer thickness, as shown in Figure 2a. The conductivity of the ink approaches 2×10^7^ S m^−1^ at 150 °C and 1×10^7^ S m^−1^ at 80 °C with increased overprints. Note that the 150 °C heating was achieved on a glass substrate since the acrylic material would deform at temperatures above 80 °C. A low-cost 250-watt IR lamp is used to cure the ink by putting the substrate under the light for five minutes after each printed layer. The maximum measured temperature of the substrate is 80 °C. This method was capable of achieving a high conductivity of 1×10^7^ S m^−1^ by ~6 overprints. From the cross-section of Scanning Electron Microscopy (SEM) images (Supplementary Information Figure S1), there are clear voids in the printed film that are subsequently filled in by the overprints. More overprints result in a dense film with higher conductivity. Via 8 overprints and IR curing cycles, the film is fairly dense and ~5-μm-thick, as seen in Figure 2b. Adhesion was a major concern for the ink, and it was found experimentally that the addition of 0.02 wt% 2-HEC solved the issue, while increasing viscosity for superior jetting. Adhesion to glass, PET, and the 3D printed materials was tested with scotch tape without any removal of the silver film. The ink is stable over the long term, as tested with a Dimatix 10-pL cartridge over five months with no observable reduction in the overall drop mass. Additionally, the printed films show no significant difference in conductivity after 10 months aging in environmental conditions (Supplementary Information Figure S2).

### Fabrication process

**Figure 3:**
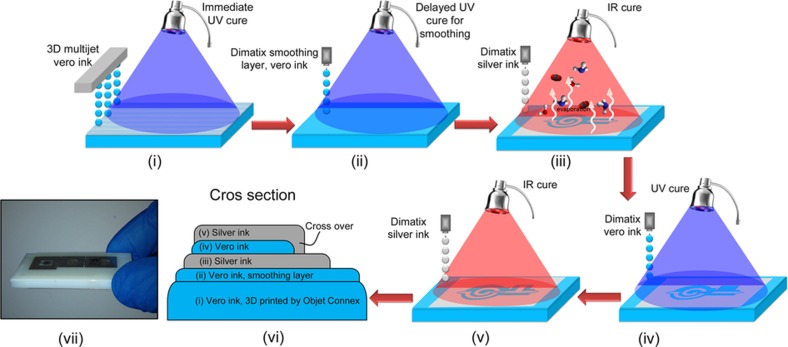
Schematic presentation of the fabrication process for multilayer inkjet-printed radio frequency electronics.

**Figure 4:**
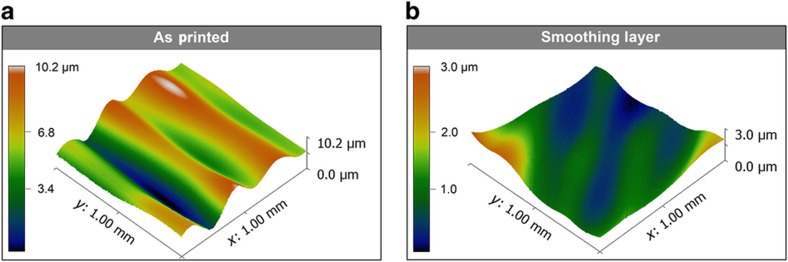
(**a**) White light interferometer of the 3D printed part showing 1.8 μm of RMS roughness. (**b**) After a smoothing layer of acrylic dielectric ink is applied, there is now 0.4 μm of RMS roughness.

A depiction of the complete inkjet process is shown in Figure 3. Two different printers were utilized, the Objet 3D multijet printer and Dimatix 2831 research printer, for convenience. The multijet printer allows quick processing of the UV material, while the Dimatix printer allows for more control over all of the print parameters and curing. It is easy to envision a single inkjet system or production line capable of performing all printing and curing. First, the UV cure material is printed with the Objet 3D printer, allowing for complex and thick shapes to be printed (i). After 3D printing, the substrate has several micrometers of surface roughness, as shown in Figure 4a. This roughness is substantial considering that the printed metal thickness itself is less than a micrometer per layer. Surface roughness is especially detrimental in RF components since it causes attenuation of the signal. The roughness issue is tackled by jetting an additional ‘smoothing’ layer of dielectric ink (Figure 4b) with the Dimatix 2831 inkjet printer and 10-pL head (ii). After deposition, the ink is allowed to spread before UV curing, and eventually, it smoothens the surface to 0.4 μm of RMS roughness. SOC ink can then be printed on this smooth layer and cured with IR heating (iii). Next, in step (iv), the dielectric ink is printed with the Dimatix, and the dielectric ink covers the silver ink and is again cured with a UV lamp. Finally, in step (v), another layer of SOC ink can be printed, creating a multilayer process where the top and bottom layers are connected with crossovers. A cross-section depiction of the process is shown in Figure 3(vi). Note that the silver and dielectric printing (iv–v) could be repeated to create several layers for more complex electronics designs. An image of a fully printed part is shown in Figure 3(vii).

One difficulty to overcome during the fabrication was the wetting of the dielectric VeroWhite ink on the silver layer. The dielectric ink spreads excessively on the solid silver layer underneath. To optimize the wetting condition, a perfluorodecanethiol (PFDT) treatment was used. This treatment has been demonstrated by Tseng *et al.* to increase the surface hydrophobicity of printed electrodes^27^. The parts are dipped in a bath of 0.28 mL PFDT with 100 mL of IPA, then rinsed in IPA and dried with nitrogen. The change can be seen in the contact angle measurement of the dielectric, as is shown in Supplementary Information Figure S3. Without treatment, the ink spreads uncontrollably on the surface (note that all images are taken 5 s upon impact). After 10 min of surface treatment, the contact angle was stable at 65 °C, and high definition patterns were possible with the dielectric ink on top of the silver. This allows for the dielectric and silver to be printed in a multilayer fashion capable of fabricating RF passives.

## Experimental

### Silver-organo-complex ink formulation

In an illustrative experiment, a 2 M ethylamine (NH_2_CH_2_CH_3_, ACS reagent) solution in methanol, which was called ‘Complexing Solution #1’, was put in a vial. 10 mL of ethanolamine (NH_2_CH_2_CH_2_OH, ACS reagent, ≥99.0%) and 10 mL of deionized (DI) water (1:1 ratio) were mixed in another vial. Formic acid (HCOOH, reagent grade, ≥95.0%) was then added to the solution in a drop-wise manner to adjust the solution to a pH of 10.5. The resulting solution was called ‘Complexing Solution #2’. In another vial, 1 g of silver acetate (CH_3_COOAg, ReagentPlus, 99%) was vortex mixed with 2 mL of complexing solution #1, 1.5 mL of complexing solution #2, and 0.5 mL of 2% 2-hydroxyethylcellulose (2% 2-HEC in water: methanol MW=90 000) at room-temperature for 30 s, resulting in a light black-colored solution. 2-HEC not only acts as a viscosifier, it also acts as an additive for adhesion of the ink to the substrate. The as-obtained solution was then kept for twelve hours to allow any particles to settle out, yielding a clear supernatant, which was decanted and filtered through a 200-nm syringe filter. This clear and transparent solution, containing approximately ~17 wt% silver, served as the silver-ethylamine-ethanolamine-formate complex-based SOC ink^25^.

### Characterization of the capacitor, inductor and filter

Capacitors were measured with an Agilent 4980A LCR meter. The leakage current was measured with a Keithley 4200-SCS.High-frequency measurements of the passive devices were taken in a two-port configuration using 500-μm-pitch Z-probes and a cascade probe station with an Agilent E8361A network analyzer. The structural properties were examined using scanning electron microscopy (FEI NovaNano FEG-SEM 630). The thickness and uniformity of printed features on substrates were measured using a surface profiler (Veeco Dektak 150) and 3D interferometry (Zygo, Newview 7300). The surface tension and viscosity of the inks were measured using a KRUSS DSA100 and Brookfield Rheometer (DV3T). Thermogravimetric analysis was performed using a TG 209 F1 analyzer (Netzsch), with a heating rate of 10 °C min^−1^ in air flow.

## Results

### Low-frequency capacitor characterization

**Figure 5:**
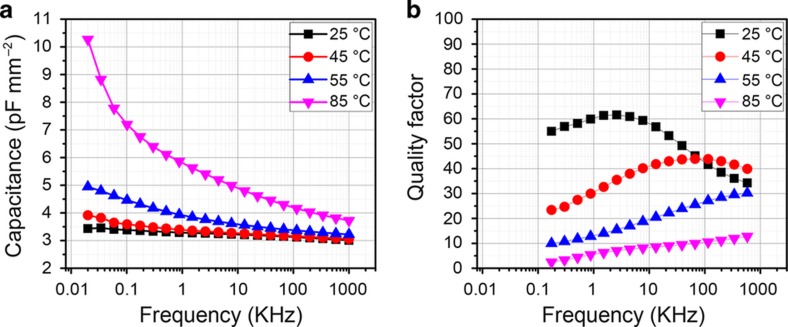
(**a**) Capacitance at low frequency as a function of temperature, (**b**) quality factor, tested at 1 V AC signal and 0 V bias condition. 11-μm-thick printed layers.

To evaluate the process, Metal Insulator Metal capacitors were first printed with the SOC ink for electrodes and the dielectric ink using the process previously described. Capacitors allow for characterization of the leakage current, dielectric, behavior and quality factor at low frequency. The printed capacitors have excellent leakage current values of approximately 1×10^−10^ A cm^−2^ at 0.08 MV cm^−1^, equivalent to 100 V across the 11-μm-thick capacitor (plotted in Supplementary Information Figure S4). The capacitors were also tested against frequency and temperature; see Figures 5a and b. While the dielectric constant in Figure 5a is relatively flat with a frequency (~3) up to 45 °C, the material shows considerable low-frequency dispersion at elevated measurement temperatures. This temperature issue can also be seen in the decrease in the quality factor shown in Figure 5b. After cooling to room temperature, the dielectric properties return to the 25 °C case.

This is a known behavior in acrylic materials, caused from dielectric relaxation at the glass transition temperature, (~50 °C). A thorough investigation of the dielectric relaxation in thin acrylic sheets has been studied by Wubbenhost *et al.*, who described this phenomenon^28^. A complete characterization of the dielectric relaxation with temperature for this material is out of the scope of this work. The important point is that the dielectric properties are sensitive to temperature and should be operated below the glass transition. Humidity impacts the capacitors, providing a ~10% normalized capacitance increase accompanied by a quality factor reduction when tested from 25% relative humidity to 85% relative humidity (Supplementary Information Figure S5). The dielectric has also been tested against voltage bias and shows ideal behavior with negligible change in capacitance value or quality factor (Supplementary Information Figure S6). The major changes in both physical and electrical dielectric properties occur at elevated temperature. However, there is no issue as long as the material is fabricated below 80 °C to avoid excessive warping and operated below 45 °C to avoid dielectric changes at the glass transition temperature.

### High frequency characterization of the capacitor, inductor and filter

The capacitor, inductor and filter were all measured at high frequencies with a vector network analyzer in a two-port configuration. Electromagnetic models of the devices were created using Ansoft high frequency structure simulator (HFSS) software with the appropriate conductivity, thickness and dielectric properties to compare with measurements. From the network analyzer, the measured *S*-parameters are de-embedded with an open-short method and are converted to *Y*-parameters at each frequency point *f*, which is a standard procedure^29,30^. The following equations are used to convert to capacitance *C*, inductance *L*, and quality factor *Q*.

(1)C=1im(4Y11+Y22−Y12−Y21)2πf

(2)L=im(4Y11+Y22−Y12−Y21)2πf

(3)Q=−(im(Y11+Y22−Y12−Y21)re(Y11+Y22−Y12−Y21))

**Figure 6:**
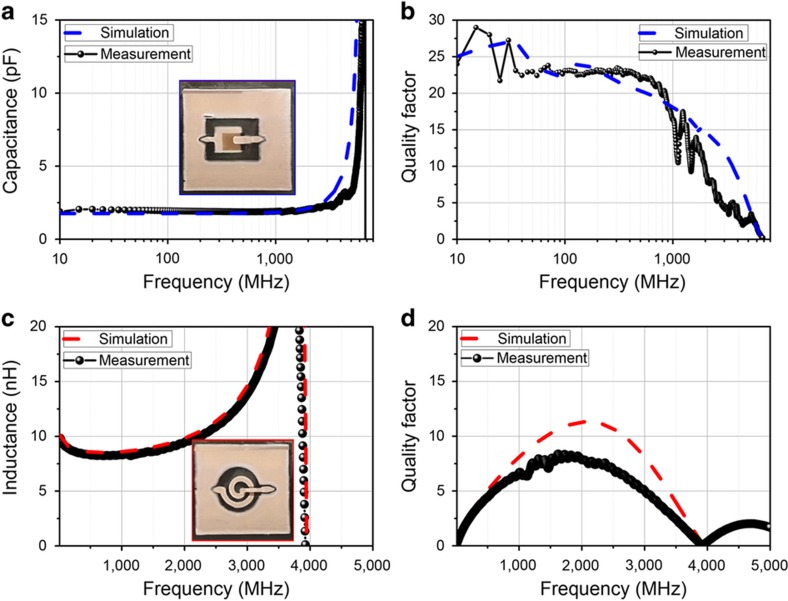
(**a**) Capacitor value measured and simulated, (**b**) quality factor (capacitor area is ~0.9 mm^2^ with an 11-μm dielectric), (**c**) inductor value measured and simulated, and (**d**) quality factor (1.5 turn inductor with an outer radius of 4 mm and 600-μm-thick lines).

Figures 6a and b show the quality factor and the capacitance value as a function of frequency. The capacitor is 2 pF and has a self-resonant frequency of 6.5 GHz. The quality factor of the capacitor starts out at 25 and drops down to zero at self-resonance as expected. The quality factor results are also consistent with the measured devices at lower frequency and 25 °C, as shown in Figure 5b. It should be noted that previous printed dielectrics have had difficulty achieving such high quality factors in the high MHz and GHz regime, typically reporting Q’s lower than 10, largely due to the loss tangent of the dielectrics used^8,9^.

An inductor has also been tested, as shown in Figures 6c and d. This 1.5 turn inductor has an outer diameter of 4 mm with a 600-μm-thick spiral, has a self-resonance at 4 GHz and is approximately 8 nH at a frequency of 1 GHz. The quality factor in simulation is slightly higher than the measured results in Figure 6d. This is likely due to there being greater resistance from the printed silver ink than simulated; the inductors are extremely sensitive to the conductivity and thickness of the printed ink. These inductors have been printed with five layers of SOC ink and show a peak quality factor of ~8. The quality factors for both the inductors and capacitors are considered state-of-the-art among inkjet-printed passives, even with the low 80 °C processing temperature^8,9^.

**Figure 7:**
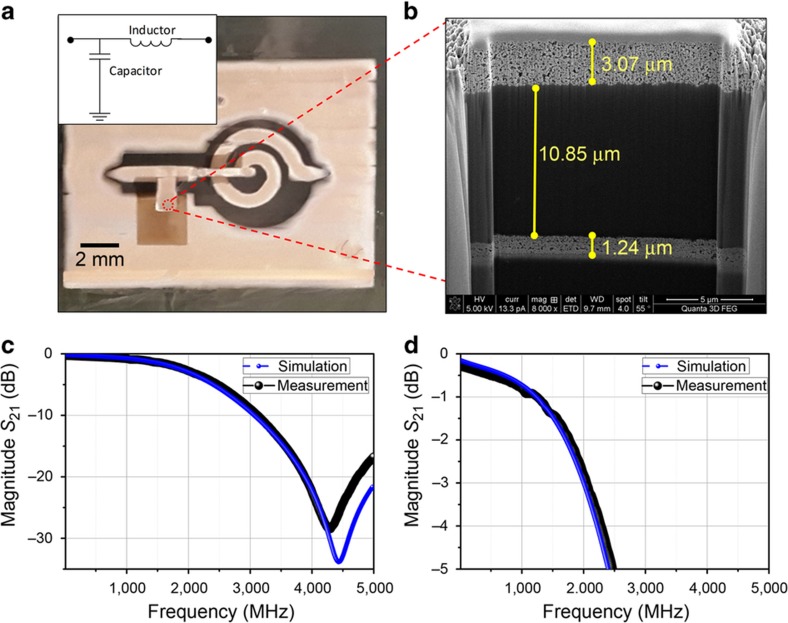
(**a**) Microscope image of the printed filter. (**b**) SEM focused ion beam cross-section image through the capacitor area, showing the thickness of the printed dielectric and the top and bottom electrodes of the filter. (**c**) Measured and simulated *S*_21_ filter response versus frequency. (**d**) Zoomed in view of the filter response.

The capacitor and inductor were implemented in a classic Butterworth low pass filter with a cutoff frequency of 2.0 GHz (Ref. 31). The Butterworth filter provides a maximally flat passband, and the low pass filter was designed in a full-wave EM simulation in Ansoft HFSS to finalize the layout. Figure 7a shows a microscopy image of the fabricated filter. There is an inset depicting the corresponding placement of the capacitor and inductor for clarity. The capacitor area is further visualized by the cross-sectional SEM image in Figure 7b. From the cut, the printed silver thickness at both layers and the 11-μm-thick dielectric spacing of the capacitor are clear. The measured frequency response of the filter in Figures 7c and d matches well with the HFSS simulation with a 3-dB cutoff at 2 GHz. There is signal rejection near 10 dB at 3 GHz and better than 20 dB at 4 GHz. The filter has no ripple in the passband, as expected, and an insertion loss of 0.8 dB at 1 GHz from the zoomed-in response of Figure 7d. The low insertion loss is excellent for a first ever fully inkjet-printed lumped element filter, especially considering the temperature constraints of 80 °C. The performance is state of the art, even compared with previous 2D distributed printed filters where nanoparticles are printed on a standard substrate, reporting insertion losses ranging from 3.6 to 10 dB (Refs. 11–15).

## Discussion

Inkjet printing is transitioning from solely a graphic arts medium into a useful fabrication tool. The ability to deposit multiple materials and the scalability of inkjet systems with hundreds of nozzles makes it possible to realize large and complex parts. A process is presented in this work in which inkjet-printed UV-cured polymer and silver ink are integrated together. A major challenge is integrating a metal with the low-temperature UV-cured plastic material. A novel SOC ink has been deployed, developed in-house, which provides a conductivity of 1×10^7^ S m^−1^ at 80 °C to combine the materials effectively. The combination of quick (5-minute) IR curing of the silver and rapid UV curing of the polymer in an ambient environment makes this an attractive method for fabrication. The capacitor and inductor exhibit state-of-the-art quality factors of ~20 and 8, respectively, in the radio frequency regime and compare well with electromagnetic simulation models. By implementing these components, a low pass filter has been fabricated with an insertion loss of 0.8 dB at 1 GHz. This is excellent considering that this is the first demonstration of a fully inkjet-printed lumped element filter. Although there is still room for improvement in terms of the conductivity of the metal, loss tangent of the dielectric, and temperature range of the printed acrylic, this is a significant step forward.

## Figures and Tables

**Table 1:** Fluid properties—SOC ink is measured at 25 °C with a jetting velocity of 10 m s^−1^

Ink Type	Viscosity (cp)	Surface T. Dyne/cm	Density (g/cc)	Drop Mass (ng)	(Oh)	(Re)	(We)	(Ca)
SOC Ink	5.9	30.7	1.17	7.0	0.21	41.6	80.0	1.9
VeroWhite Dielectric Ink	20.0	30.2	1.1	9.6	0.75	10.4	62	6.0

VeroWhite dielectric ink is measured at 60 °C with a jetting velocity at 9 m s^−1^ velocity. Both inks utilize a Dimatix 10-pL DMC cartridge with a 21-μm-diameter nozzle.

Ca, Capillary; Oh, Ohnesorge; Re, Reynolds; Surface T., Surface Tension; We, Weber.
