# Supplementary material for: Fully inkjet-printed microwave passive electronics
Source: Microsyst Nanoeng. 2017 Jan 30;3:16075. doi: 10.1038/micronano.2016.75 (PMC6444987; doi:10.1038/micronano.2016.75)
Supplement: Supplementary Information [file micronano201675-s1.pdf]

Supplementary file

Fully inkjet-printed microwave passive electronics

Garret McKerricher[1,]*, Mohammad Vaseem[1,]* and Atif Shamim[1]

Microsystems & Nanoengineering (2017) **3,** 16075; doi:10.1038/micronano.2016.75; Published online: 30 January 2017

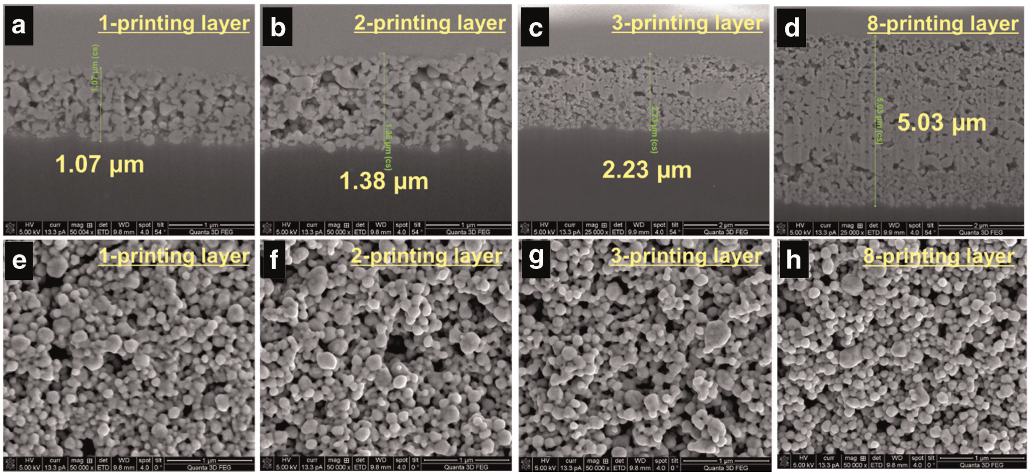

Figure S1 (**a**–**d**) Cross section focused ion beam and SEM profiles of the printed silver film. (**e**–**h**) SEM images of the corresponding surfaces.

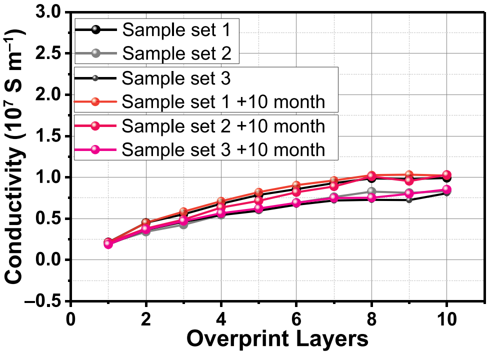

Figure S2 Conductivity measurements of three samples originally after fabrication and after 10 months storage in ambient environment.

[1]King Abdullah University of Science and Technology (KAUST), IMPACT Lab, Computer, Electrical and Mathematical Sciences and Engineering (CEMSE) Division, Thuwal 23955-6900, Saudi Arabia.
Correspondence: Garret McKerricher (garret.mckerricher@kaust.edu.sa)
*These authors contributed equally to this work.

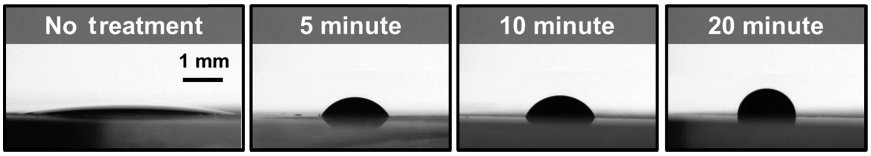

Figure S3 Contact angle measurements showing the effect of perfluorodecanethiol treatment on the spreading of the dielectric ink ontop of a solid printed silver layer.

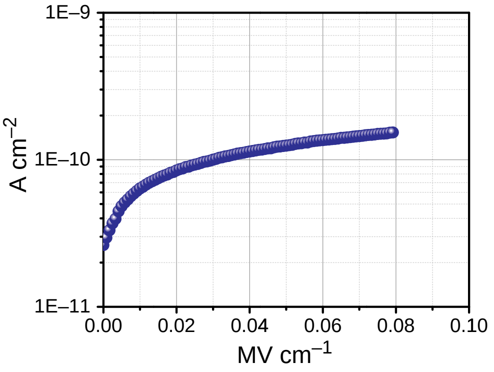

Figure S4 Leakage current of a typical fully printed capacitor (tested to 100 V ~ 0.08 MV cm^{-1}) Dielectric thickness 11 μm.

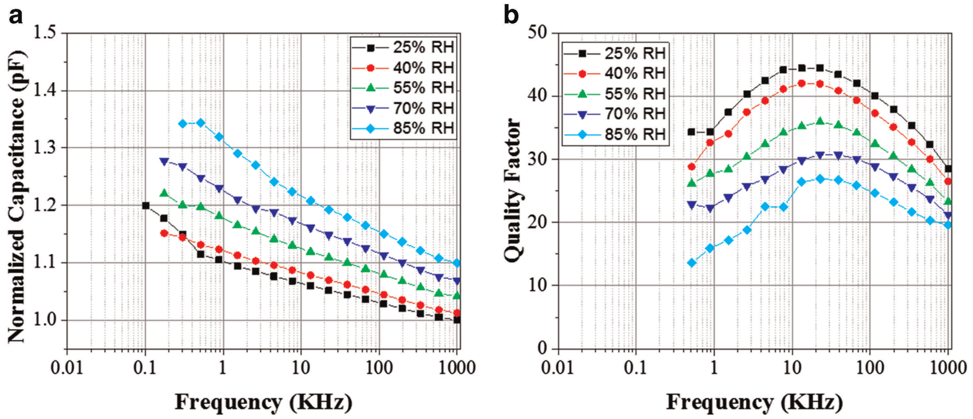

Figure S5 Capacitor tested after 10 month storage and as a function of relative humidity. (**a**) Capacitance and (**b**) quality factor.

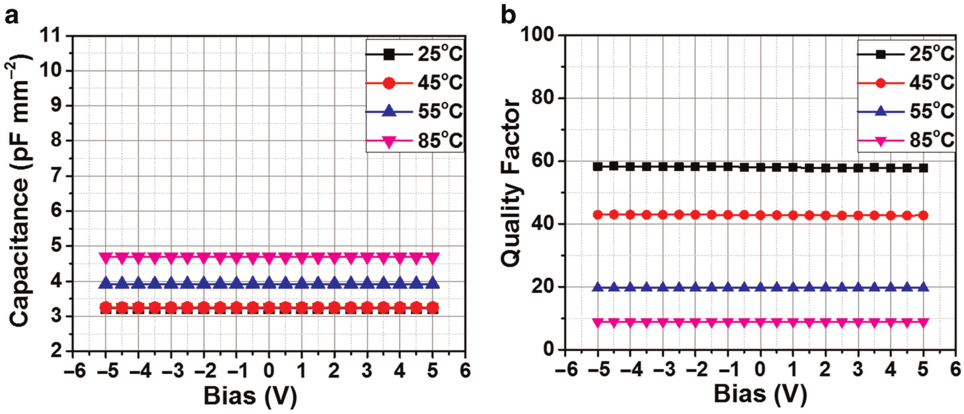

Figure S6 (**a**) Capacitance versus bias. (**b**) Quality factor. Measured with a 1 V AC signal and 10 KHz frequency.
